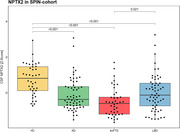# The assessment of synaptic dysfunction in neurodegenerative disorders by a novel single molecular array for Neuronal pentraxin 2

**DOI:** 10.1002/alz.095251

**Published:** 2025-01-09

**Authors:** Mathias Sauer, Bárbara Fernandes Gomes, Henrik Zetterberg, Kaj Blennow, Ann Brinkmalm, Olivia Belbin, Daniel Alcolea, Juan Fortea, Oskar Hansson, Johanna Nilsson, Nicholas J. Ashton

**Affiliations:** ^1^ Department of Psychiatry and Neurochemistry, Institute of Neuroscience and Physiology, The Sahlgrenska Academy, University of Gothenburg, Mölndal, Gothenburg Sweden; ^2^ Sant Pau Memory Unit, Hospital de la Santa Creu i Sant Pau, Biomedical Research Institute Sant Pau, Universitat Autònoma de Barcelona, Barcelona Spain; ^3^ Memory Clinic, Skåne University Hospital, Malmö Sweden

## Abstract

**Background:**

Synaptic dysfunction is a prominent feature in neurodegenerative disorders, associating with cognition. In contrast to most synaptic proteins assessed in cerebrospinal fluid (CSF), Neuronal pentraxin 2 (NPTX2), essential for synaptic plasticity, exhibits downregulation neurodegenerative disorders through mass spectrometry (MS) methods. While MS‐based assays hold promise, their constraints hinder widespread clinical adoption. Thus, our study aimed to develop a novel single molecular array (Simoa) for quantifying NPTX2 levels in CSF.

**Method:**

**Homebrew** NPTX2 was validated for the Simoa platform using commercial antibodies (EPR15618, ERP24020‐38; Abcam) in 2uL of CSF. After validation, we performed a study in Alzheimer’s disease (AD) and healthy controls (HC) from the BioFINDER pilot cohort (n = 99 participants; 48.5%AD; mean age 76.2 years [7.0 SD]). As a further validation, we quantified NPTX2 in the Sant Pau Initiative on Neurodegeneration (SPIN) cohort with 199 patients (58 AD, 51 HC, 60 Lewy body dementia (LBD) and 41 behavioral variant of frontotemporal dementia (bvFTD)). The mean age was 69.5 (11.4 SD) and both cohorts had an even distribution of sex. We examined group differences by the Wilcoxon rank sum test (corrected with Dunn‐test) and associations between the biomarkers and MRI, Tau PET, amyloid‐β (Aβ) PET and MMSE were determined by linear regression, adjusting for age and sex.

**Result:**

In BioFINDER, Fold change (FC) in NPTX2 was significantly decreased in AD (median [95%CI], 0.85[0.65‐1.00]; p = 0.005) as compared to HC. NPTX2 showed a significant association in the full cohort with MMSE (β = 5.46, *p*<0.001), cortical thickness (β = 0.18, *p*<0.001) and Tau PET (β = ‐0.46, *p* = 0.002), but not with Aβ PET. In the SPIN cohort, the FC for neurodegenerative groups were significantly decreased as compared to HC (AD, 0.58[0.52‐0.79]; *p*<0.001; LBD, 0.66[0.56‐0.81]; *p*<0.001; and bvFTD, 0.50[0.33‐0.61]; *p*<0.001), with AUCs of 0.77(AD, 95%CI = 0.68‐0.86), 0.73(LBD, 0.63‐0.83) and 0.86(bvFTD, 0.78‐0.94). NPTX2 showed a significant association with MMSE (β = 2.10, *p* = 0.01) in the full cohort. Further results will explore NPTX2 in blood.

**Conclusion:**

This novel ultra‐sensitive immunoassay confirms the decrease of CSF NPTX2 in neurodegenerative diseases and a significant relationship to cognitive decline. This approach could streamline and increase the assessment of synaptic dysfunction in neurodegenerative diseases, providing valuable insights for disease management and treatment strategies.